# *Magnusiomyces capitatus* bloodstream infection in a patient with acute monocytic leukemia: A rare case report

**DOI:** 10.1016/j.mmcr.2025.100718

**Published:** 2025-07-16

**Authors:** Tian Qi, Mi Zhou, Xingchun Chen, Heping Xu, Weihua Pan, Xinying Ding

**Affiliations:** a Department of Dermatology, Shanghai Key Laboratory of Molecular Medical Mycology, Second Affiliated Hospital of Naval Medical University, Shanghai, China; b Children's Hospital of Soochow University, Suzhou, China; c The People's Hospital of Guangxi Zhuang Autonomous Region, Nanning, China; d The First Affiliated Hospital of Xiamen University, Xiamen, China; e Zibo First Hospital, Zibo, China

**Keywords:** *Magnusiomyces capitatus*, Bloodstream infection, Acute leukemia, Antifungal resistance, Molecular diagnostics

## Abstract

A 26-year-old male with relapsed acute monocytic leukemia (AML-M5) in China developed *Magnusiomyces capitatus* fungemia during consolidation chemotherapy. Diagnosis was confirmed by MALDI-TOF MS and ITS sequencing. Antifungal susceptibility revealed resistance to echinocandins but sensitivity to amphotericin B. Serial (1,3)-β-D-glucan monitoring correlated with disease progression. This case highlights diagnostic challenges and therapeutic dilemmas in regions lacking epidemiological data.

## 1 Introduction

*Magnusiomyces capitatus* (formerly *Geotrichum capitatum* or *Saprochaete capitata*), an environmental saprophyte, can be isolated from environmental sources such as wood, soil, in animals (including bovine mastitis and poultry faeces), and has been found in dishwashers [1]. As an opportunistic pathogen, it primarily infects immunocompromised individuals, particularly those with hematological malignancies such as acute myeloid leukemia (AML) or acute lymphoblastic leukemia (ALL) [2,3]. This dimorphic yeast has increasingly been recognized as an emerging etiologic agent of invasive fungal infections (IFI). Notably, definitive identification requires molecular methods due to limitations of conventional microscopy, when combined with limited therapeutic options, this diagnostic challenge contributes to the pathogen's characteristically high mortality rates [1,4,5].

We report a case of a 26-year-old male with AML - M5 who developed *M. capitatus* bloodstream infection during consolidation chemotherapy. In China, fewer than ten cases of invasive *M. capitatus* infections have been reported to date, leading to significant gaps in clinical understanding. This case report comprehensively documents the clinical progression, diagnostic approaches, and therapeutic strategies of this rare infection, providing valuable insights for future clinical practice and research.

## 2 Case presentation

A 26-year-old male with a 5-year history of acute monocytic leukemia (AML-M5) was admitted to our hospital for consolidation chemotherapy. Five years ago, the patient developed recurrent fevers (up to 39.0 °C) with occasional chills, gum swelling, and sore throat. In June 2018, blood tests at our hospital showed markedly abnormal results: WBC 57.7 × 10^9^/L, Hb 87g/L, PLT 22 × 10^9^/L, and 93 % blasts. Suspecting leukemia, the patient was admitted for further testing. Bone marrow studies and genetic analysis confirmed *EVI1* and *FLT3-ITD* mutations, leading to a diagnosis of acute monocytic leukemia (AML-M5). He underwent multiple cycles of chemotherapy, including IA regimen (idarubicin + cytarabine) and high-dose cytarabine. Post-chemotherapy genetic re-evaluation showed negative results for both *EVI1* and *FLT3-ITD* gene mutations. However, due to the unavailability of a fully matched bone marrow donor, the patient continued consolidation chemotherapy at our institution.

On admission (Day 0), the patient presented with a fever of 38.5 °C. Physical examination revealed mild pallor but no signs of active infection. Laboratory tests demonstrated severe pancytopenia (WBC: 0.48 × 10^9^/L, ANC: 0.02 × 10^9^/L, Hb: 83g/L, PLT: 33 × 10^9^/L) and elevated lactate dehydrogenase (308 U/L). Nucleic acid tests for Epstein - Barr virus (EBV), Cytomegalovirus (CMV), Mycoplasma pneumoniae, Chlamydia pneumoniae, Influenza A and B, as well as SARS-CoV-2, were negative. Fungal (1,3)-β-D-glucan (G-test) and galactomannan (GM-test) were also negative (G-test: <40 pg/mL, GM-test: <0.1 μg/L). Procalcitonin (PCT) was mildly elevated (0.202 ng/mL). Plain CT scan of the lungs and mediastinum showed no abnormalities, but splenomegaly was observed.

**Fig. 1:**
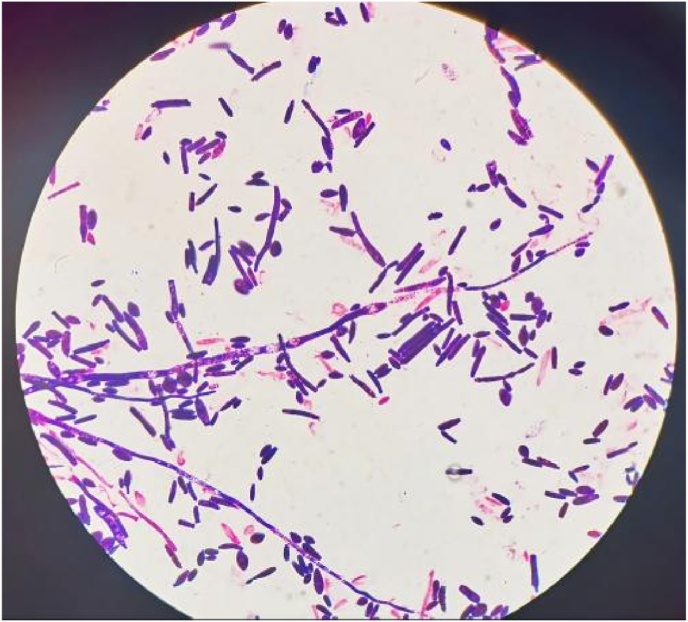
Direct Microscopy of Blood Culture: Microscopic examination of colonies revealing fungal spores and hyphae.

**Fig. 2:**
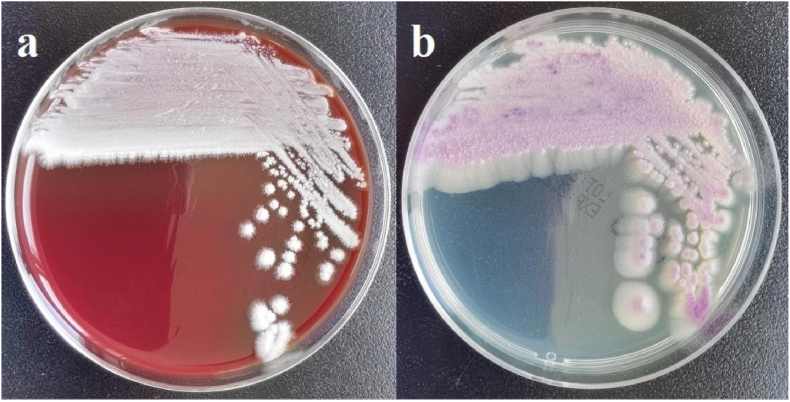
Culture characteristics: (a) Incubated at 35 °C for 6 days on Sabouraud Dextrose agar, demonstrating increased colony size (diameter:3–4mm) and more pronounced roughness. (b) After incubation at 35 °C for 6 days on Candida chromogenic agar, flat, purple-pink colonies with serrated edges were observed.

On Day +5, the patient developed persistent fever (peak temperature: 40.2 °C). Blood cultures drawn on Day +6 grew yeast-like organisms within 22 hours. The samples were inoculated onto Sabouraud dextrose agar (SDA) plates and *Candida* chromogenic plates. Fungal spores were visible in direct smear staining of blood culture and plate colonies[Fig. 1]. On SDA, white, serrated, dull, and rough colonies formed, and on Candida chromogenic plates, flat, purple-pink colonies were observed [Fig. 2]. The isolate was identified as *Magnusiomyces capitatus* through combined molecular methods: ITS sequencing (GenBank: MH037208.1) provided preliminary identification, and MALDI-TOF MS analysis definitively confirmed the species identity, overcoming limitations of ITS in discriminating closely related species. The raw data of ITS sequencing and MALDI-TOF MS analysis have been provided as Supplementary Files.

Antifungal susceptibility testing revealed resistance to echinocandins (micafungin, caspofungin, anidulafungin: MIC = 2 μg/mL) and susceptibility to amphotericin B (MIC = 1 μg/mL) and azoles (voriconazole MIC = 0.25 μg/mL; posaconazole MIC = 0.25 μg/mL; fluconazole MIC = 4 μg/mL). During hospitalization, serial G-test monitoring was performed. The G-test level rose sharply from <40 pg/mL on Day +1–2810.5 pg/mL on Day +10, coinciding with fungemia progression. Following amphotericin B initiation (150 mg/day from Day +10), levels declined progressively to 508.8 pg/mL on Day +17, normalizing to 47.7 pg/mL at discharge[Table 1]. The patient received intravenous amphotericin B (150 mg/day for 2 days, titrated to 350 mg/day for 13 days). However, due to the emergence of characteristic amphotericin B-associated infusion-related reactions (IRRs), manifested as pyrexia, rigors, and transient hypotension, the daily dose was administered through divided infusions - 150 mg and 200 mg administered at distinct time intervals within the same treatment day - to mitigate adverse effects while maintaining therapeutic efficacy. Despite therapy, the patient experienced intermittent fever and persistent neutropenia. PCT levels increased to 2.18 ng/mL, suggesting ongoing systemic inflammation. After 15 days of treatment, the patient reported subjective improvement in fever but opted for discharge against medical advice. Recent clinical follow-up confirmed the patient's survival; however, his general health status remains suboptimal, requiring ongoing medical monitoring.

**Table 1:** Serial (1,3)-β-D-glucan dynamics correlated with disease progression and antifungal therapy.

Time	G-test(pg/ml)	Therapy(Amphotericin B)
Day+1	40	Not administered
Day+6	713.4	Not administered
Day+10	2810.5	150mg/d(Day+10-Day+11)
Day+17	508.8	350mg/d(Day+12-Day+20)
Discharge	47.7	Discontinued

## 3 Discussion

*Magnusiomyces capitatus* is an emerging opportunistic pathogen historically concentrated in Mediterranean and Central Europe among patients with hematological malignancies [3,6,7]. In China, fewer than ten invasive *M. capitatus* infections have been documented to date, based on our comprehensive review of Chinese (Wanfang, CNKI, VIP) and international (PubMed, Web of Science) databases (Girmenia et al. reported only 2 Chinese cases among 99 globally) [6,8]. This highlights its expanding geographical footprint and underscores epidemiological gaps in non-endemic regions. Similar to European cohorts, our patient exhibited classic risk factors, including prolonged neutropenia (ANC <0.1 × 10^9^/L) and chemotherapy-induced mucosal disruption [9]. Notably, while Mediterranean studies report mortality rates exceeding 60 % [10], our patient achieved clinical stabilization with amphotericin B, emphasizing the prognostic impact of early diagnosis and species-directed therapy. Enhanced surveillance is urgently needed to define regional risk profiles.

Conventional microscopy may misidentify *M. capitatus*. MALDI-TOF MS provided definitive speciation, overcoming limitations of ITS sequencing in resolving *M. capitatus* from *M. clavatus* due to intra-genomic heterogeneity [11]. This aligns with ESCMID/ECMM guidelines advocating mass spectrometry or multi-locus sequencing for rare yeasts [1]. G-test elevation (<40 → 2810.5 pg/mL) paralleled fungemia progression, with post-therapy decline (47.7 pg/mL) confirming its monitoring utility. However,the initial negative G-test underscores the need for multimodal diagnostics during early infection.

The isolate exhibited intrinsic echinocandin resistance (MIC = 2μg/mL) and amphotericin B susceptibility (MIC = 1μg/mL), consistent with known resistance mechanisms [12,13]. Liposomal amphotericin B remains first-line per guidelines, though persistent neutropenia likely attenuated efficacy, as neutrophil recovery is critical for clearance [14]. To mitigate infusion-related reactions, we employed split-dosing (150 mg + 200 mg/day), demonstrating a feasible strategy for tolerance management. Adjunctive approaches (e.g., granulocyte transfusions or voriconazole combination) warrant study in refractory cases, balancing efficacy and toxicity.

This study has several limitations. First, environmental sampling to trace the infection source was not performed, leaving exposure routes speculative. Second, the lack of long-term follow-up limits insights into relapse risks or late complications. Finally, antifungal susceptibility testing followed CLSI M27-A3 criteria, which lack validated breakpoints for *M. capitatus*, necessitating cautious interpretation. Future research should prioritize establishing clinical breakpoints and exploring genomic determinants of echinocandin resistance.

This case illustrates diagnostic and therapeutic challenges of *M. capitatus* fungemia. Clinicians in non-endemic regions should consider rare fungi in neutropenic patients with persistent fever. Early MALDI-TOF/MS identification, serial β-D-glucan monitoring, and tailored antifungal regimens are pivotal for improving outcomes. Multidisciplinary efforts to address epidemiological gaps and optimize therapies are imperative.

## CRediT authorship contribution statement

**Tian Qi:** Writing – original draft. **Mi Zhou:** Writing – review & editing. **Xingchun Chen:** Writing – review & editing. **Heping Xu:** Writing – review & editing. **Weihua Pan:** Writing – review & editing. **Xinying Ding:** Writing – review & editing.

## Funding information

There are none.

## Conflict of interest

There are no conflicts of interest.
