# Supplementary material for: Magnusiomyces capitatus bloodstream infection in a patient with acute monocytic leukemia: A rare case report
Source: Med Mycol Case Rep. 2025 Jul 16;49:100718. doi: 10.1016/j.mmcr.2025.100718 (PMC12284553; doi:10.1016/j.mmcr.2025.100718)
Supplement: Multimedia component 1 [file mmc1.pdf]

Autof ms1000鉴定报告

基本信息

样品名： H3_1.spectrum

样品点： H3

样品描述： ---

检验人员： Administrator

生成时间： 2025年07月04日 08时11分

鉴定结果： 头状大孢酵母菌 / Magnusiomyces capitatus 9.513

质谱图

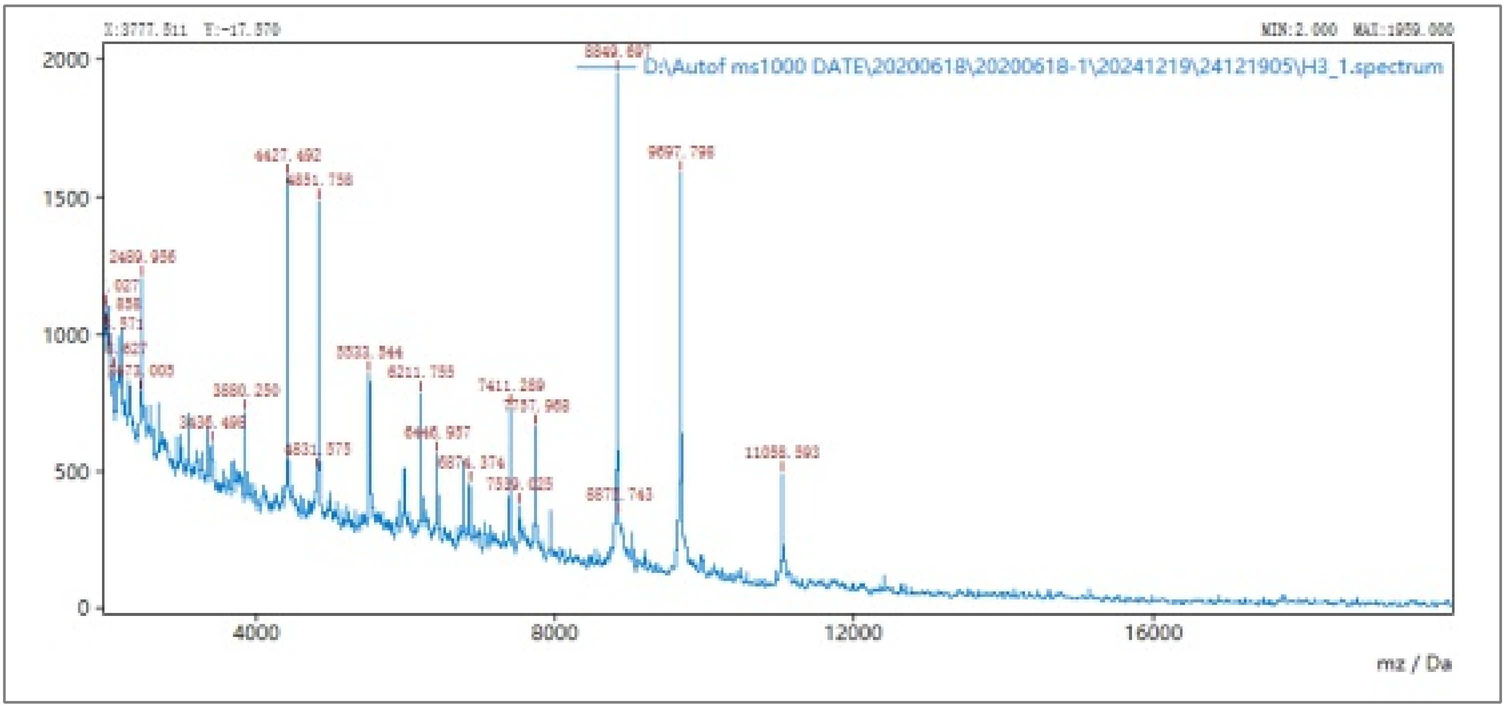

详细结果

序号	结果	得分
1	头状大孢酵母菌 / Magnusiomyces capitatus	9.513
2	头状大孢酵母菌 / Magnusiomyces capitatus	9.294
3	头状大孢酵母菌 / Magnusiomyces capitatus	9.245
4	头状大孢酵母菌 / Magnusiomyces capitatus	9.115
5	头状大孢酵母菌 / Magnusiomyces capitatus	9.044
6	头状大孢酵母菌 / Magnusiomyces capitatus	8.836
7	头状大孢酵母菌 / Magnusiomyces capitatus	8.363
8	头状大孢酵母菌 / Magnusiomyces capitatus	8.273
9	头状大孢酵母菌 / Magnusiomyces capitatus	7.387
10	头状大孢酵母菌 / Magnusiomyces capitatus	7.042
