# Supplementary material for: Magnusiomyces capitatus bloodstream infection in a patient with acute monocytic leukemia: A rare case report
Source: Med Mycol Case Rep. 2025 Jul 16;49:100718. doi: 10.1016/j.mmcr.2025.100718 (PMC12284553; doi:10.1016/j.mmcr.2025.100718)
Supplement: Multimedia component 2 [file mmc2.pdf]

北京睿博兴科生物技术有限公司
RuiBio BioTech

睿博兴科

靶向 DNA 测序鉴定微生物科研报告书

客户姓名	姚俊飞	课题组	安图生物
样本编号	ITS-240711205	生产编号	YP30203752
扩增基因名称	ITS	收样日期	20240717
实验员	吴玉蒙	审核	翟晓红
主检实验室：	北京睿博兴科生物技术有限公司研发部		

项目	检测方法	结果
靶向 DNA 测序鉴定微生物	基因测序法	Magnusiomyces capitatus（MH037208.1）100.00%

结果分析：

基因扩增可见单一清晰目的条带，测序结果经 blast 比对显示，与其他菌株序列相似度为：

1：Magnusiomyces capitatus（MH037208.1）100.00%

2：Magnusiomyces capitatus（MH037206.1）100.00%

附序列：

Seq：

TATATTTTATATTACTTTGTGGACATTTGGTTGAATTTACATGTTTTATTACAAAAAATTAATTATAATTAAAAAAATAAT
TTTAAGAAAACCTCCAACAACGGATCTCTTGGTTCCCAGATCGATGAAGAGCGCAGCGAATTGCGAAATGTGATGT
GTATTGCAGTGAATCATCAATTCTTGAACGCACATGGCACCCCTTTTAGGGGTATGCTTGTATGAGGGTGTTTAATAT
GAAATTGCTTTGGCTTTTTTTAAATAAATGGTTTTTCAAATTGTTACTAATAGACGAAAGAATCAGTGCAACAAGCTG
TGTTGAATCTTTCATTAAATCTTTTAGTTAACTACTTTAACTATTTGCACCTCATATCAAGCAAGACTACCCGCTGAAC
TTAAGCATATCAATAAGTCGGAGGAAAA

注：对于某些属的细菌或者真菌，因靶向基因过于保守而难以鉴定到种，我们会给出鉴定到属的结果，并报告最可能的种，之后请结合形态、生化、MS 等其他方法进行综合判断。

北京睿博兴科生物技术有限公司 报告专用章

报告日期

2024 年 7 月 18 日

本检测不排除假阴性或假阳性。
